# The Prevalence and Correlates of HIV and Undiagnosed Infection among Men Who Have Sex with Men in Hanoi, Vietnam: Findings from a Cross-sectional, Biobehavioral Study

**DOI:** 10.3389/fpubh.2016.00275

**Published:** 2016-12-19

**Authors:** Nga Thi Thu Vu, Martin Holt, Huong Thi Thu Phan, Lan Thi La, Gioi Minh Tran, Tung Thanh Doan, John de Wit

**Affiliations:** ^1^Centre for Social Research in Health, UNSW Australia, Sydney, NSW, Australia; ^2^Institute of Preventive Medicine and Public Health, Hanoi Medical University, Hanoi, Vietnam; ^3^Vietnam Administration for HIV/AIDS Prevention and Control, Ministry of Health, Hanoi, Vietnam; ^4^Hanoi Centre of HIV/AIDS Prevention and Control, Hanoi, Vietnam; ^5^Center for Community Health Promotion, Hanoi, Vietnam; ^6^Utrecht University, Utrecht, Netherlands

**Keywords:** HIV prevalence, amphetamine type stimulants, men who have sex with men, undiagnosed HIV, Hanoi—Vietnam

## Abstract

**Introduction:**

Men who have sex with men (MSM) are a key population for HIV infection in Vietnam, and the use of amphetamine type substances (ATS) is prevalent and possibly increasing in this population. The reported analysis examines the association between ATS use before or during sex and HIV infection among MSM in Hanoi, Vietnam.

**Methods:**

This cross-sectional study of 210 MSM was conducted in Hanoi, Vietnam, in late 2014. Men tested for HIV and answered questions about demographic characteristics, sexual sensation seeking, depression, belief in HIV prevention strategies, homosexuality-related stigma and discrimination, recent accessing of HIV prevention services, sexual behaviors and ATS, and other drug use behaviors. We performed logistic regression to assess correlates of HIV infection.

**Results:**

HIV prevalence was 6.7% (14/210), and 85.7% (12/14) of HIV-positive men were not aware of their HIV status. Of the 210 participants, 10.5, 2.9, and 3.8% of men had used methamphetamine, amphetamine, and ecstasy before or during sex in the last 3 months. In multivariable analysis, HIV infection was associated with recent sex-related methamphetamine use [adjusted odds ratio (AOR): 5.03, 95% confidence interval (CI): 1.35–18.68], engaging in recent sex work (AOR: 3.55, 95% CI: 1.07–11.75), and homosexuality-related perceived stigma (AOR: 2.32, 95% CI: 0.98–5.47).

**Conclusion:**

Findings underscore the importance of integrating methamphetamine use interventions into HIV prevention services and scaling-up of gay-friendly, non-stigmatizing HIV testing services for MSM in Hanoi. We recommend the routine assessment of ATS use and undiagnosed infection in this population.

## Introduction

The HIV epidemic was first described in Vietnam in the early 1990s (1), and in the last 30 years, the epidemic has spread to different parts of the country. Since 1999, HIV has been reported in all provinces and cities of Vietnam (2). Although men who have sex with men (MSM) have been recognized as a key population affected by HIV in different parts of the world (3), in Vietnam, they were only identified as a priority population for HIV prevention in the last decade (4), despite warning signs in the early 2000s that HIV was spreading in this population (5, 6).

A study conducted only in Ho Chi Minh City in 2004 reported an HIV prevalence of 8% in a sample of MSM recruited in MSM-specific venues (7). The first, national integrated biological behavioral surveillance (IBBS) conducted in 2006 reported that the HIV prevalence among MSM in Ho Chi Minh City and Hanoi was 5 and 9%, respectively (8). In the second IBBS conducted in 2009, HIV prevalence exceeded 10% in Hanoi (19.8%) and Ho Chi Minh City (14.3%), as well as in Haiphong (16.6%), the country’s third largest city (9), suggesting HIV prevalence had increased substantially among MSM in major cities across Vietnam. A more recent study found an HIV prevalence of 14.8% among MSM in Ho Chi Minh City (10). A better understanding of the individual, social, and structural factors that affect HIV risks is, therefore, needed to prevent further transmission of HIV in the MSM population in Vietnam.

Previous studies have found that HIV infection among MSM in Vietnam is associated with younger age, lower education and injecting drug use (7, 11), a higher number of recent sexual partners (5, 7), and sex work and inconsistent condom use (5). MSM in Vietnam have consistently reported low levels of condom use with casual and regular male partners (8, 9).

Amphetamine type stimulants (ATS) are commonly used drugs by MSM, and their use has been found to be associated with condomless anal intercourse (CAI) and HIV infection (12). A recent meta-analysis found a significant association between methamphetamine and amphetamine use and HIV infection across 35 studies (13). However, most previous studies have been conducted in high-income countries, and evidence regarding the association between ATS use and HIV infection among MSM is lacking in low- and middle-income countries (13), particularly Vietnam. It was reported that ATS use, particularly methamphetamine use is prevalent among MSM in Vietnam (14). The relationship between ATS use and HIV infection among MSM in Vietnam, however, remains to be assessed.

In Vietnam, homosexuality remains socially stigmatized (15), despite recent activism to protect the rights of lesbian, gay, bisexual, and transgender people. Previous international research underscores that homosexuality-related stigma and discrimination are associated with a higher risk of depression (16, 17). Depression has been found to be highly prevalent among MSM, particularly HIV-positive men (18, 19), and is considered a major health issue among MSM (20). Furthermore, homosexuality-related stigma, discrimination, and depression have been found to be associated with engaging in HIV-related risky behaviors such as CAI (21–23), drug use (24, 25), having sex while on drugs (26), less awareness and underutilization of HIV prevention services (27–29), and experiencing less benefit from participating in HIV prevention interventions (27).

Previous studies of HIV infection among MSM in Vietnam have mainly focused on individual-level covariates rather than examining social and structural barriers to HIV prevention, such as homosexuality-related stigma and discrimination. Evidence from previous studies indicates that sexual sensation seeking is associated with an increased likelihood of CAI among MSM (30–33) and that sexual sensation seeking can modify or strengthen the association between alcohol or drug use and HIV-related sexual behaviors (34, 35). However, the effect of sexual sensation seeking on HIV risk among MSM in Vietnam has not been previously investigated.

Previous research in Vietnam has found relatively high rates of CAI between MSM (8, 9), and international research has found that MSM may use various strategies to reduce HIV transmission risk during CAI, such as serosorting (having sex with partners of the same perceived HIV status), strategic positioning (HIV-negative men being insertive during CAI), withdrawal before ejaculation, or limiting CAI to HIV-positive partners who are virally suppressed (36, 37). However, belief in the efficacy of these strategies has not been previously assessed among Vietnamese MSM.

To improve HIV prevention among MSM in Vietnam, we sought to assess the prevalence of HIV and undiagnosed infection among MSM in Hanoi and examined factors associated with HIV infection, including homosexuality-related stigma and discrimination, depression, sexual sensation seeking, belief in the efficacy of risk reduction strategies, and drug use, particularly ATS use.

## Materials and Methods

Participants included in this analysis were part of a larger convenience sample of 303 MSM recruited into a cross-sectional, community-based study in Hanoi, Vietnam, from September to October 2014. The study received approval from the Human Research Ethics Committee of the University of New South Wales, Australia, and from the Institutional Review Board of the Hanoi School of Public Health. A description of the study has been previously published (14). In short, the study was conducted in collaboration with the Hanoi Center for HIV/AIDS Prevention and Control (Hanoi PAC), the Center for Community Health Promotion (CHP), and MSM community-based organizations (CBOs). MSM are a relatively hidden population in Vietnam, and developing a randomized sampling frame is impossible. Convenience sampling was hence used to recruit participants. First, staff and outreach workers of collaborating organizations referred potential participants to the study. Additionally, peers of collaborating CBOs referred men from their social networks. Finally, participated men were asked to invite their peers who might be interested in the study.

Men were eligible to participate if they were 18 years or older at the time of the study, reported having anal sex with at least one man in the previous 3 months, had a good command of the Vietnamese language, and provided consent. Men were screened for eligibility and were interviewed after provided written consent. Upon interview completion, men were informed about optional HIV testing.

Men who chose to be tested were referred to an on-site testing team provided by Hanoi PAC. Separate written consent was obtained for HIV testing. Venous blood samples were collected by technicians from Hanoi PAC. Blood samples were assigned a unique, anonymous ID code enabling linkage to participants’ questionnaires (and for participants to get results). Due to resource constraints, we could only offer 210 free HIV tests to participants.

### Confidentiality and Privacy

Being identified as a MSM or drug user in Vietnam may result in social stigma and discrimination (38). As such, MSM participants, particularly those who use drugs, may underreport sexual and drug use behaviors. We took several steps to mitigate this potential bias. First, all interviewers were trained to be supportive and non-judgmental of MSM. Second, MSM participants were not asked to provide personal details that could reveal their identity. Third, all interviews were conducted in private rooms. Finally, all field staff signed a confidentiality agreement.

### HIV Testing and Status

Blood samples were tested for HIV at the Hanoi PAC laboratory, following Vietnamese Ministry of Health guidelines. All blood samples were screened for HIV by the Murex HIV Ag/Ab combination assay (DiaSorin S.p.A., Italy). Samples that were reactive during screening were tested again using confirmatory tests (Serodia^®^ HIV, Fujirebio, Japan; Determine™ HIV1/2, Alere Medical, USA). The main outcome variable was HIV status as confirmed by testing (HIV negative or HIV positive). We also referred to participants’ self-reported HIV status (HIV negative, untested/unknown, or HIV positive) to identify participants who tested HIV positive but were unaware of their infection. The self-report information collected through the questionnaire has previously been described (14) and contained the questions described below.

### Demographic Characteristics

We asked participants about their self-reported HIV status, sexual orientation, age, place of birth, education, occupation, and monthly income.

### Sexual Sensation Seeking

We adapted a measure of sexual sensation seeking for a Vietnamese sample (39). Ten items assessed the propensity to seek out exciting and novel sexual experiences, for instance “I like wild, uninhibited sexual encounters.” Participants provided their answers on 5-point scales, ranging from 1 (not at all like me) to 4 (very much like me). Internal consistency of the items was sufficient (Cronbach’s α = 0.72). Item scores were averaged, with higher scores indicating more sexual sensation seeking.

### Depression

This was assessed with the Patient Health Questionnaire 9 scale, which has been used with MSM in other studies (40, 41). The scale consists of nine items, such as “in the last two weeks, how often have you had trouble falling or staying asleep, or sleeping too much?” Participants provided their answers on a scale ranging from 0 (not having the problem at all) to 3 (having the problem nearly every day). The items had good internal consistency (Cronbach’s α = 0.80). Participants were categorized as having a depressive disorder if they had a score equal or larger than 10, as previously described (42).

### Belief in HIV Prevention Strategies

Participants were asked how effective they thought different strategies were in preventing HIV transmission: antiretroviral treatment of HIV, HIV-negative men taking the insertive (top) position during anal sex, and withdrawal before ejaculation. Answer options ranged from 1 (totally disagree) to 4 (totally agree). Belief in the efficacy of each HIV prevention method was dichotomized into disagreement (scores 1 and 2) versus agreement (scores 3 and 4).

### Perceived Stigma and Discrimination

We adapted a scale to measure enacted (experienced) homosexuality-related stigma, perceived (anticipated) homosexuality-related stigma, and self-stigma (internalized homophobia) (22). Responses were given on 4-point scales, with anchors depending on the questions. The adapted scale encompassed eight items pertaining to enacted homosexuality-related stigma, for example “how often have you lost a job or career opportunity due to your engaging in homosexual activities” (1 = never, 4 = often); 10 items measuring perceived homosexuality-related stigma, for instance “many people are unwilling to accept homosexual individuals” (1 = completely disagree, 4 = completely agree); and eight items measuring internalized homophobia, for example “sometimes you wish you were not gay/bisexual/transgender” (1 = totally disagree, 4 = totally agree). In this study, the scale had good internal consistency (Cronbach’s α = 0.74). Mean scores were calculated for each sub-scale; higher scores indicated higher levels of stigma.

### Recent Accessing of HIV Prevention Services

We asked participants if they had recently tested for HIV (i.e., at least once in the last 12 months) and if they had recently received safe sex counseling (i.e., at least once in the last 12 months).

### Sexual Behaviors

We asked about the gender of sexual partners and age at first sex with men and women. Participants were asked about ever engaging in sex work (selling sex), recent sex work (in the last 3 months), their number of regular and casual male partners in the last 3 and 12 months, and the use of condoms during anal sexual intercourse with regular and casual male partners. Because numbers of different types of sexual partners were skewed, we undertook logarithmic or square root transformations of these variables, as appropriate. Any CAI was defined as not or inconsistently using a condom during anal sex (assessed for regular and casual male partners in the previous 3 months).

### ATS and Other Drug Use

Participants were asked questions about ever having used ATS, alcohol, and other substances (i.e., ketamine, erectile dysfunction medications, and amyl nitrite “poppers”), any recent use of these substances (in the last 3 months) and any use of these substances before or during sex in the last 3 months. Answers to these questions were dichotomized (any use versus none).

### Data Analysis

Descriptive and correlational statistical analyses were performed using STATA version 13.1 (StataCorp, College Station, TX, USA). We report frequencies and percentages for categorical variables and medians with interquartile ranges (IQR) for continuous variables. We tested bivariate associations between laboratory-confirmed HIV status and potential covariates using logistic regression. Factors associated with the outcome variable at *p* < 0.25 were subsequently entered into a multivariate regression model to identify independent covariates. We used a stepwise procedure as previously suggested (43) to develop the final multivariate model with independent covariates, which retained statistical significance (*p* < 0.05).

## Results

A total of 222 men were referred to the study as potential participants, among whom nine men did not meet selection criteria, two men chose not to complete an interview, and one man refused to be tested. As such, the following analysis includes 210 men who completed an interviewer-administered questionnaire and agreed to be tested for HIV and release their HIV test result. We compared the sociodemographic characteristics of men who did and did not undertake HIV testing (analysis not shown). Men who tested for HIV and consented to release their HIV test results were significantly younger, had lower incomes, and were more likely to be students or self-employed. Men who did and didn’t undertake HIV testing were similar in terms of place of birth, education level, sexual orientation, and perceived HIV status.

### Sample Characteristics

Table 1 presents the demographic characteristics of the 210 MSM who participated. The majority of the sample reported being homosexual (73.3%), 22.4% bisexual, and the remainder (3.8%) heterosexual or other sexual orientation. The median age of the sample was 22.7 years (IQR: 20.6–25.5); 90.0% were younger than 30 years. The majority of men had a college or university education (57.9%), 30.1% had high school or vocational training, and only 12.0% had secondary or lower education. Approximately 30.0% of the sample was students, 9.0% were unemployed, and the remainder had office-based jobs, service jobs, or were in self-employed, casual, or freelance jobs. Participants had a median monthly income of 5 million Vietnamese Dong (approximately US$228) (IQR: 3.0–8.0 million). The median sexual sensation-seeking score was 2.5 (IQR: 1.4–3.5). A minority of men (14.3%) were categorized as having depression. In relation to belief in different HIV prevention strategies, 9.5% men believed in the effectiveness of HIV treatment as prevention, 28.6% believed in the safety of being insertive during sex, and 38.6% men believed that withdrawal was effective. The median score regarding homosexuality-related enacted stigma was 1.1 (IQR: 1.0–2.4); scores for perceived stigma and internalized homophobia were 4.0 (IQR: 1.20–5.0) and 3.4 (IQR: 1.0–4.6), respectively.

**Table 1:** **Participant characteristics**.

	Frequency	Percent (95% CI)
**HIV status confirmed by lab tests (*N* = 210)**		
Negative	196	93.3 (89.1–96.3)
Positive	14	6.7 (3.7–10.9)

**Perceived HIV status (on enrollment) (*N* = 210)**		
HIV positive	2	0.9 (0.1–3.4)
HIV negative	110	52.4 (45.4–59.3)
Tested but didn’t know the result	62	29.5 (23.4–36.2)
Never previously tested	36	17.1 (12.3–22.9)

**Sexual identity (*N* = 210)**		
Homosexual	154	73.3 (66.8–79.2)
Bisexual	47	22.4 (16.9–28.6)
Heterosexual and other	9	4.3 (2.0–8.0)

**Age [median and interquartile ranges (IQR)] (*N* = 210)**	22.7 (20.6–25.5)	
<20	38	18.1 (13.1–24.0)
20–29	151	71.9 (65.3–77.9)
30–39	14	6.7 (3.7–10.9)
≥40	7	3.3 (1.4–6.7)

**Place of birth (*N* = 210)**		
Hanoi	86	41.0 (34.2–47.9)
Other provinces	124	59.0 (52.1–65.8)

**Education (*N* = 209)**		
Primary and lower school	25	12.0 (7.9–17.1)
High school and vocational training	63	30.1 (24.0–36.9)
College and university	121	57.9 (50.9–64.7)

**Occupation (*N* = 210)**		
Student	66	31.4 (25.2–38.2)
Office-based job	35	16.7 (11.9–22.4)
Service job	38	18.1 (13.1–24.0)
Self-employed/freelance	52	24.8 (19.1–31.2)
Unemployed	19	9.0 (5.5–13.8)

**Median monthly income (IQR) (*N* = 210)**	5.0 (3.0–8.0)	
Income <3 million VND	37	17.6 (12.7–23.5)
3 million VND ≤ income <5 million VND	62	29.5 (23.4–36.2)
Income ≥5 million VND	111	52.9 (45.9–59.8)

**Median sexual sensation-seeking score (IQR) (*N* = 210)**	2.5 (1.4–3.5)	

**Depression (*N* = 210)**	30	14.3 (9.9–17.8)

**Belief in HIV prevention strategies (*N* = 210)**		
Treatment as prevention	20	9.5 (5.9–14.3)
Being insertive during anal sex	60	28.6 (22.6–35.2)
Withdrawal	81	38.6 (32.0–45.5)

**Median score for homosexuality-related stigma and discrimination (IQR) (*N* = 210)**		
Enacted stigma	1.1 (1.0–2.4)	
Perceived stigma	4.0 (1.2–5.0)	
Internalized homophobia	3.4 (1.0–4.6)	

**Any HIV test in the last 12 months (*N* = 210)**	93	44.3 (37.5–51.3)

**Received any safe sex counseling in the last 12 months (*N* = 210)**	110	52.4 (45.4–59.3)

### Sex and Drug Use Behaviors

Table 2 presents HIV-related sex and drug use behaviors. Approximately 63% men self-reported having sex with men only; the remainder reported having sex with both men and women. The median age of first homosexual sex was 19.0 (IQR: 18.0–21.0), and the median age of first heterosexual sex was 18.0 (12.0–30.0). Of the 210 participants, 73.8% reported any recent CAI with male partners (66.2% with regular partners and 32.4% with casual partners). The median number of regular male sexual partners in the last 3 months was 1 (IQR: 1.0–80.0), and the median number of casual male sexual partners in the last 3 months was 3.0 (IQR: 1.0–100.0). Of the 210 participants, 26.2% reported having ever engaged in sex work and 21.4% reported recent sex work. One in five participants (22.9%) reported ever having used methamphetamine, 14.8% reported recent use, and 10.5% reported recent sex-related use. The corresponding rates for amphetamine use were 7.6, 2.9, and 2.9%, respectively. Rates for ecstasy they were 20.0, 8.6, and 3.8%. Men reported higher levels of alcohol use: 90.0% had ever drunk alcohol, 76.2% had recently consumed alcohol, and 39.5% reported sex-related alcohol use in the last 3 months.

**Table 2:** **Sexual and drug use behaviors**.

	Frequency	Percent (95% CI)
**Type of sexual partners (*N* = 210)**		
Male sexual partners only	132	62.9 (55.9–69.4)
Both male and female sexual partners	78	37.1 (30.6–44.1)

**Median age at first homosexual sex (*N* = 210) (IQR)**	19.0 (18.0–21.0)	

**Median age at first heterosexual sex (*N* = 78) (IQR)**	18.0 (12.0–30.0)	

**Any condomless anal intercourse (CAI) with male partners in the last 3 months (*N* = 210)**	155	73.8 (67.3–79.6)

**Regular male sexual partners**		
Median number of regular male sexual partners in the last 3 months (*N* = 196) (IQR)	1 (1–80)	
Any CAI with regular male sexual partners in the last 3 months (*N* = 210)	139	66.2 (59.4–72.6)

**Casual male sexual partners**		
Median number of casual male sexual partners in the last 3 months (*N* = 128) (IQR)	3 (1–100)	
Any CAI with casual male sexual partners in the last 3 months (*N* = 210)	68	32.4 (26.1–39.1)

**Ever engaged in sex work (*N* = 210)**	55	26.2 (20.4–32.7)

**Engaged in sex work in the last 3 months (*N* = 210)**	45	21.4 (16.1–27.6)

**Lifetime use of alcohol and other drugs (*N* = 210)**		
Alcohol	189	90.0 (85.1–93.7)
Amphetamine (speed)	16	7.6 (4.4–12.1)
Amyl nitrite (poppers)	11	5.2 (2.6–9.2)
Cannabis	35	16.7 (11.9–22.4)
Ecstasy	42	20.0 (14.8–26.1)
Erectile dysfunction medication	15	7.1 (4.1–11.5)
Heroin	12	5.7 (3.0–9.8)
Ketamine	16	7.6 (4.4–12.1)
Methamphetamine	48	22.9 (17.4–29.1)
Sleeping pills	8	3.8 (1.7–7.4)

**Any alcohol and ATS use in the last 3 months (*N* = 210)**		
Alcohol	160	76.2 (69.8–81.8)
Amphetamine (speed)	6	2.9 (1.1–6.1)
Ecstasy	18	8.6 (5.2–13.2)
Methamphetamine	31	14.8 (10.3–20.3)

**Any alcohol and ATS use before or during sex in the last 3 months (*N* = 210)**		
Alcohol	83	39.5 (32.9–46.4)
Amphetamine (speed)	6	2.9 (1.1–6.1)
Ecstasy	8	3.8 (1.7–7.4)
Methamphetamine	22	10.5 (6.7–15.4)

*CI, confidence interval; IQR, interquartile range*.

### HIV Testing Results

Fourteen men out of 210 [6.7%, 95% confidence interval (CI): 3.7–10.9] tested HIV-antibody positive (see Table 1) and 12 of these 14 HIV-positive men (85.7%) were not aware of their HIV status. Just over half (52.4%) of all participating men had ever tested for HIV, and just under half (46.6%) did not know their HIV status, including 29.5% who had ever tested for HIV but did not know their test result and 17.1% who had never tested for HIV. Out of the 210 men who were tested, approximately 10 men returned for their results, including one man who was previously undiagnosed with HIV.

### Correlates of HIV Infection

The results of bivariate and multivariate analyses of associations between HIV infection and other covariates are presented in the Table 3. Potential independent covariates identified in bivariate analysis included occupation, sexual sensation seeking, belief in the safety of being insertive during sex as a HIV prevention strategy, homosexuality-related perceived stigma, engaging in recent sex work, number of regular male sexual partners, any recent CAI with casual male sexual partners, recent methamphetamine or amphetamine use before or during sex, and having a HIV test in the last 12 months. In the final multivariate logistic regression model, HIV infection was more likely among MSM who reported recent sex work or the use of methamphetamine before or during sex. Additionally, HIV infection was marginally associated with homosexuality-related perceived stigma.

**Table 3:** **Bivariate and multivariate analysis of associations with HIV infection (*N* = 210)**.

	COR (95% CI)	*p*	AOR (95% CI)	*p*
**Age group (in years)**		0.415		
<20	1			
20–29	1.14 (0.24–5.51)	0.87		
≥30	3.0 (0.46–19.59)	0.251		

**Education**		0.01		0.843
Primary and lower school only	1		1	
High school and above	5.61 (1.52–20.79)		1.20 (0.20–7.34)	

**Occupation**		0.04		
Student or office-based job	1		1	
Service job	4.95 (1.12–21.85)	0.035	0.30 (0.01–8.65)	0.485
Self-employed/freelance	4.26 (1.02–17.80)	0.047	0.28 (0.01–6.82)	0.435
Unemployed	1 (omitted)^a^			

**Monthly income**		0.307		
<3 million VND (US$163.4)	1			
3 million–4.9 million VND	0.28 (0.05–1.58)	0.148		
≥5 million VND	0.64 (0.18–2.26)	0.49		

**Sexual orientation**		0.857		
Homosexual	1			
Bisexual	0.89 (0.24–3.32)	0.858		
Heterosexual and other	1 (omitted)^a^			

**Had major depression in the last 2 weeks**	2.56 (0.51–12.74)	0.252		

**Sexual sensation seeking**	4.59 (1.17–17.96)	0.023	2.27 (0.47–11.08)	0.31

**Belief in the safety of withdrawal as a prevention strategy**	0.88 (0.28–2.72)	0.82		

**Belief in the effectiveness of HIV treatment as a prevention strategy**	1 (omitted)^a^			

**Belief in the safety of being insertive during sex as a prevention strategy**	0.40 (0.09–1.83)	0.235	0.25 (0.05–1.32)	0.102

**Homosexuality-related enacted stigma**	1.48 (0.24–9.20)	0.672		

**Homosexuality-related perceived stigma**	1.99 (0.92–4.31)	0.081	2.32 (0.98–5.47)	0.054

**Homosexuality-related homophobia**	0.78 (0.43–1.42)	0.425		

**Had male partners only versus both male and female partners**	0.94 (0.30–2.90)	0.909		

**Engaged in sex work (selling sex) in the last 3 months**	4.16 (1.38–12.56)	0.012	3.55 (1.07–11.75)	0.038

**Number of regular male sexual partners**	0.24 (0.04–1.63)	0.145	1.38 (0.08–23.31)	0.822

**Number of casual male sexual partners**	1.34 (0.81–2.20)	0.255		

**Any condomless anal intercourse (CAI) with regular sexual partners in the last 3 months**	1.95 (0.53–7.22)	0.319		

**Any CAI with casual sexual partners in the last 3 months**	3.02 (1.00–9.09)	0.049	1.76 (0.29–10.58)	0.537

**Any CAI in the last 3 months**	1.32 (0.36–4.93)	0.676		

**Methamphetamine use before or during sex in the last 3 months**	5.85 (1.76–19.44)	0.004	5.03 (1.35–18.68)	0.016

**Amphetamine use before or during sex in the last 3 months**	8.0 (1.33–48.15)	0.023	1.73 (0.14–21.29)	0.668

**Ecstasy use before or during sex in the last 3 months**	1 (omitted)^a^			

**Drinking alcohol before or during sex in the last 3 months**	1.58 (0.53–4.68)	0.41		

**Having at least one HIV test in the last 12 months**	0.48 (0.15–1.59)	0.229	1.0 (0.21–4.68)	0.999

**Receiving any safe sex counseling in the last 12 months**	0.66 (0.22–1.98)	0.463		

*COR, crude odds ratio; AOR, adjusted odds ratio*.

*^a^One of the cells contained a value of 0 and was excluded from the analysis*.

## Discussion

To the best of our knowledge, this is the first reported analysis of an association between a broad range of important structural, personal dispositions, and behavioral factors and HIV infection among MSM in Vietnam. While we found that MSM who reported recently using amphetamine or ecstasy before or during sex were not more likely to have an HIV positive test result, MSM who self-reported the recent use of methamphetamine before or during sex had a fivefold increased likelihood of HIV infection, compared with MSM who did not use methamphetamine for sex. These findings are similar to those of a recent meta-analysis (13), as well as recent empirical research, finding higher rates of methamphetamine and/or amphetamine use among diagnosed HIV-positive MSM compared to HIV-negative MSM (44–47). In our study, we also found that men who engaged in recent sex work were more likely to test positive for HIV. Together, our findings suggest that men who use ATS for sex and men who engage in sex work should be targeted as priority groups for HIV prevention in Hanoi, Vietnam. We also suggest that interventions for methamphetamine use are made available for MSM in Vietnam and be incorporated into current HIV prevention activities. Furthermore, future periodic surveillance of HIV among MSM in Vietnam would benefit from the inclusion of measures of ATS use and its association with sexual risk behaviors. Such research would contribute to an increasing understanding of the complex relationships between drug use, sexual behaviors, and HIV infection among Vietnamese MSM that can guide HIV prevention.

We found a relatively moderate prevalence of HIV (6.7%) among MSM recruited in Hanoi, lower than that found in previous government-run surveillance studies (6, 8). Our convenience sample did not include any MSM who reported injecting drug use, so we may have under-recruited men who are at higher risk of HIV. As such, generalizing our findings to the broader community of MSM in Hanoi should therefore be undertaken with caution. Strikingly, we found the majority of HIV-positive men (12 out of 14) indicated that they were HIV-negative or did not know their HIV status. To the best of our knowledge, no previous studies have reported the level of undiagnosed HIV among MSM in Vietnam (7, 9, 11, 48), and our results suggest that many HIV-positive MSM in Hanoi may be unaware of their infection. Some of the HIV-positive men in our study may have chosen not to reveal that they had already been diagnosed when interviewed, because of reticence or fear about disclosing their status. Previous studies in high-income countries, while reporting lower levels of undiagnosed HIV (49, 50), have found that men with undiagnosed HIV report more risky sexual and drug use behaviors than HIV-negative men (49). We also found a borderline association between homosexuality-related perceived stigma and HIV infection in our analysis. Therefore, promoting accessible HIV testing services, for example, MSM-run, community-based, HIV testing services, is recommended, because they could encourage HIV testing as well as returning for HIV test results (51). Additionally, as little is known about undiagnosed HIV among MSM in Vietnam, future research could assess the extent of undiagnosed HIV and its correlates in different parts of the country.

We did not find a significant association between HIV infection and any form of CAI, despite previous research establishing CAI as a key risk factor for HIV infection in MSM (52). Since the sample size for this analysis was relatively small, power may have been insufficient to detect an association between CAI and HIV infection. Alternatively, the lack of variance between HIV status groups may reflect a ceiling effect of high levels of CAI in both HIV-positive and non-HIV-positive MSM. Because CAI with male sexual partners was so common in the sample (73.8%) and the level of undiagnosed HIV was strikingly high, we recommend an intensified promotion of safe sex among MSM in Hanoi.

Previous studies have found that depression can be highly prevalent among MSM and is more likely higher among diagnosed, HIV-positive MSM (41, 53). However, we did not find an association between depression and HIV infection in our sample. This may be because we found a relatively low prevalence of depression in the sample, and the majority of men who had HIV were unaware of their infection. We also did not find an independent association between sexual sensation seeking and HIV infection. However, like other researchers, we have previously found a positive relationship between sexual sensation seeking and CAI (30–33).

Our study had several limitations. Since MSM are a hidden population in Vietnam, we used a convenience sampling approach, as a result of which our findings may not be representative of the broader MSM population in Hanoi. Our sample is more likely to be representative of young MSM in urban settings in Vietnam who are students or self-employed and have lower incomes. As MSM are a hidden population in Vietnam, previous studies have also used a variety of non-random convenience sampling methods and, as in many high income countries, no randomized samples of MSM have been recruited in Vietnam (8, 9, 38, 54–56). Also, although we tried to eliminate reporting bias during the interview process, our findings might reflect underreporting of sexual and drug use behaviors and other socially sensitive issues. Additionally, men who engaged in transactional sex were disproportionately affected by HIV (57). As one fifth of the sample engaged in sex work, assessing sex practices with their sexual partners would have been useful but no such assessment was included in our study.

## Conclusion

We found a moderate prevalence of HIV infection among MSM in Hanoi, Vietnam, and most of the men who tested HIV-positive seemed to be unaware of infection. HIV infection was associated with sex-related methamphetamine use and engagement in sex work. Our analytical and descriptive findings point to several recommendations. For HIV prevention, we recommend an integration of interventions for methamphetamine use into HIV prevention, an intensified promotion of safe sex, and implementation of community-based, MSM-run, or MSM-friendly HIV testing services in Hanoi, Vietnam. In research, we recommend the routine assessment of ATS use in national HIV surveillance and research to enable analysis of trends in ATS use and associations with sexual behaviors. We also recommend further study of men’s beliefs and practices with respect to various harm reduction strategies. Finally, we urge examination of the extent of undiagnosed HIV infection in MSM in different parts of Vietnam.

## Author Contributions

NV led the development of the research protocol and data collection tools, undertook data collection and data analysis, wrote the initial draft of the manuscript, and prepared the final manuscript. JW and MH guided and supervised the development of the research protocol, data collection and analysis, and contributed to the writing of the manuscript. HP, LL, GT, and TD provided advice and guidance on the research protocol and data collection and contributed to the manuscript.

## Conflict of Interest Statement

The authors declare that the research was conducted in the absence of any commercial or financial relationships that could be construed as a potential conflict of interest. The reviewer ZW and handling Editor declared their shared affiliation, and the handling Editor states that the process nevertheless met the standards of a fair and objective review.
